# Paromomycin production from *Streptomyces rimosus* NRRL 2455: statistical optimization and new synergistic antibiotic combinations against multidrug resistant pathogens

**DOI:** 10.1186/s12866-019-1390-1

**Published:** 2019-01-18

**Authors:** Asmaa A. Ibrahim, Ghadir S. El-Housseiny, Khaled M. Aboshanab, Mahmoud A. Yassien, Nadia A. Hassouna

**Affiliations:** Department of Microbiology and Immunology, Faculty of Pharmacy, Ain shams University, Organization of African Unity St. Abbassia, POB: 11566, Cairo, Egypt

**Keywords:** Paromomycin synergistic combinations, *Streptomyces rimosus*, Response surface methodology, 2-dexoystreptamine-aminocyclitol aminoglycoside antibiotics (2DOS-ACAGAs)

## Abstract

**Background:**

Response surface methodology (RSM) employing Box-Behnken design was used to optimize the environmental factors for the production of paromomycin, a 2 deoxystreptamine aminocyclitol aminoglycoside antibiotic, (2DOS-ACAGA) from *Streptomyces (S.) rimosus* NRRL 2455. Emergence of bacterial resistance caught our attention to consider the combination of antimicrobial agents. The effect of paromomycin combination with other antimicrobial agents was tested on some multiple drug resistant isolates. To the best of our knowledge, this is the first report on optimization of paromomycin production from *S. rimosus* NRRL 2455. A Quadratic model and response surface method were used by choosing three model factors; pH, incubation time and inoculum size. A total of 17 experiments were done and the response of each experiment was recorded. Concerning the effect of combining paromomycin with different antimicrobial agents, it was tested using the checkerboard assay against six multidrug resistant (MDR) pathogens including; *Pseudomonas (P.) aeruginosa* (2 isolates), *Klebsiella (K.) pneumoniae*, *Escherichia (E.) coli*, methicillin sensitive *Staphylococcus aureus* (MSSA) and methicillin resistant *Staphylococcus aureus* (MRSA). Paromomycin was tested in combination with ceftriaxone, ciprofloxacin, ampicillin/sulbactam, azithromycin, clindamycin and doxycycline.

**Results:**

The optimum conditions for paromomycin production were a pH of 6, an incubation time of 8.5 days and an inoculum size of 5.5% *v*/v using the optimized media (soybean meal 30 g/L, NH_4_CL 4 g/L, CaCO_3_ 5 g/L and glycerol 40 ml/L), 28 °C incubation temperature, and 200 rpm agitation rate that resulted in 14 fold increase in paromomycin production as compared to preliminary fermentation level using the basal medium. The tested antibiotic combinations showed either synergistic effect on paromomycin activity on most of the tested MDR pathogens (45.83%), additive effect in 41.67% or indifferent effect in 12.5%.

**Conclusion:**

RSM using multifactorial design was a helpful and a reliable method for paromomycin production. Paromomycin combination with ceftriaxone, ciprofloxacin, ampicillin/sulbactam, azithromycin, clindamycin or doxycycline showed mostly synergistic effect on certain selected clinically important MDR pathogens.

**Electronic supplementary material:**

The online version of this article (10.1186/s12866-019-1390-1) contains supplementary material, which is available to authorized users.

## Background

Aminoglycosides are a group of antibiotics (streptomycin, neomycin, paromomycin and tobramycin) either produced naturally from *Streptomyces* spp. or *Micromonospora* spp. or semi-synthesized in vitro [1–3]. They reveal antimicrobial activity against a broad spectrum of several microorganisms, including Gram-positive and Gram-negative bacteria, mycobacteria and protozoa [1]. Aminoglycosides are chemically stable and have a synergistic effect with other antibacterial drugs [2]. Among these, paromomycin, neomycins, kanamycins, and gentamicins are members of the therapeutically most relevant aminoglycoside subclass of the 2-deoxystreptamine-aminocyclitol aminoglycoside antibiotics (2DOS-ACAGAs). The biosynthetic gene clusters of the respective antibiotics have been isolated, annotated and analyzed however, not all genes have biochemically analyzed [3]. *S. rimosus* subsp. *paromomycinus* NRRL 2455, is a producer of paromomycin, a 2DOS-ACAGA with broad spectrum activity against Gram-negative and most Gram-positive bacteria especially *Staphylococcus* strains particularly those resistant to oxytetracycline, erythromycin or carbomycin [4]. Paromomycin was found to be effective and safe in the treatment of certain life threatening protozoal infections such as visceral leishmaniasis [5]. Fortunately, paromomycin is unique among the available drugs as it possesses the combination of high antiamoebic, antibacterial activity and low oral toxicity. So it was recommended to be a useful therapeutic substance [6].

Unfortunately, the production of antibiotics by *Streptomyces* is not of constant property. The production can be increased or completely lost if the conditions of the fermentation process or the fermentation media itself changed [7].Therefore, both the composition of the medium and the environmental factors of the producing organism greatly influence the antibiotic biosynthesis. To accomplish maximum production of the antibiotic by any producer strain, it is essential to optimize the nutritional and environmental conditions. A statistical optimization plan based on response surface methodology (RSM) is commonly utilized to simplify the optimization process [8]. This approach helps the industry to design the best media containing substrates of lower costs and to use the most favorable environmental conditions for improved antibiotic production. Aminoglycosides are chosen to be a drug of choice for a number of diseases however their toxicity is the main reason for the decrease in their use in the past 30 years. Approaches made to overcome their toxicity, especially once daily administration, made aminoglycosides a safer decision. Aminoglycosides are a powerful choice against multiple drug-resistant pathogens [9].Increasing resistance in recent surveillance to all currently available antibiotics, including carbapenems, cephalosporins, penicillins, fluoroquinolones, and aminoglycoside is particularly important in focusing attention on usage of combination therapy [10]. When optimal doses are used appropriately, combination therapy may maximize its clinical outcomes, especially in the presence of antibacterial resistance [11]. It was found to be more beneficial to use a combination of antibiotics than monotherapy. One of the therapeutically useful combinations that showed synergism is a cell wall-active agents with an aminoglycosidic aminocyclitol and this combination has been proposed in the guidelines of several medical associations [12, 13]. Therefore, in this study, our aim was to enhance the paromomycin production by *S. rimosus* NRRL 2455 through nutritional and environmental optimization using multi-factorial design and RSM and to show the effect of paromomycin combination with other antibiotics against some clinically relevant MDR pathogens.

## Materials and methods

### Microorganisms

*S. rimosus* subsp*. paromomycinus* NRRL 2455 (paromomycin producer; kindly supplied from NRRL, USA) was cultured in tryptic soy broth (TSB) and stored in TSB containing 50% glycerol at − 20 °C [14, 15]. *Staphylococcus aureus* ATCC 25923, a standard strain used for antimicrobial susceptibility testing and six MDR clinical bacterial isolates were recovered from sputum specimens collected from patients at Sadr Al-Abbassia Hospital, Cairo, Egypt. The study was approved by the hospital Ethics Committee and faculty of Pharmacy ethical committee Nr. 227 where both informed and written consents were obtained from the patients after explaining the purpose of the study. These MDR clinical isolates were, *P. aeruginosa* (isolates PS4 and PS25), *K. pneumoniae* (isolate KP13), *E. coli* (isolate EC10) and methicillin-resistant *Staphylococcus aureus* (MRSA isolate SA36) and methicillin-sensitive *Staphylococcus aureus* (MSSA, isolate SA41). The respective isolates were cultured in Luria–Bertani broth (LB broth) [16] and stored in LB broth containing 20% glycerol at − 20 °C or cultured on nutrient agar slants at 37 °C for and subcultured every 2–3 days or whenever necessary. Antimicrobial susceptibility pattern of the six MDR clinical isolates is shown in Table 1. Kirby-Bauer disc diffusion method was used to determine the antimicrobial susceptibility testing of the collected pathogens [17]. The minimum inhibitory concentration (MIC) was performed in triplicate according to CLSI guidelines [18, 19]. Accordingly, the MDR isolates are those resistant to three or more classes of antimicrobials [20].

**Table 1:** Antimicrobial susceptibility pattern of the six MDR clinical isolates

IsisIsolate code	AK	AMX	AMC	CFR	CXM	CRO	FEP	CIP	LEV	DOX	MEM	CXT	AZM	CLR	ERY	CLI	FOX
PS4	R	R	R	R	R	R	R	R	R	S	S	R	S	S	I	R	Nd
PS25	S	R	R	R	R	R	R	S	S	S	S	I	S	S	I	R	Nd
KD13	S	R	S	R	R	R	R	R	R	I	S	R	S	S	R	R	Nd
EC10	R	R	R	R	R	R	R	R	I	I	S	I	R	I	R	R	Nd
SA36	S	R	R	R	R	R	R	R	R	S	S	R	R	R	R	S	R
SA41	S	R	R	R	R	R	R	R	R	S	I	R	R	R	R	R	S

Ant Antibiotic abbreviations: *AMK* amikacin, *AMX* amoxicillin, *AMC* amoxicillin-clavulanic acid, *CFR* cefadroxil, *CXM* cefuroxime, *CRO* ceftriaxone, *FEP* cefepime, *CIP* ciprofloxacin, *LVX* levofloxacin, *DOX* doxycycline, *MEM* meropenem, *SXT* trimethoprim-sulfamethoxazole, *AZM* azithromycin, *CLR* clarithromycin, *ERY* erythromycin, *CLI* clindamycin, *FOX* cefoxitin, *nd* non determined

### Culture media

The basal medium for *S. rimosus* subsp. *paromomycinus* was TSB [15]. For optimizing paromomycin production by the respective strain, it was grown in different microbial growth media as shown in Table 2. The growth and production of the paromomycin in each medium was determined after 6 days of incubation at 28 °C.

**Table 2:** Different culture media used for optimization of paromomycin production

Media composition	A1^a^	A2^b^	A3^c^	A4^d^	A5^e^	A6^f^	A7	A8	A9	A10	A11^g^	A12^h^
soy bean meal (g/L)	15	15	----^i^	–	–	30	30	–	–	30	–	–
yeast extract(g/L)	–	1	3	10	–	–	–	10	3	–	–	–
Peptone(g/L)	–	–	–	15	10	–	–	15	–	–	–	–
Tryptone(g/L)	–	–	5	–	–	–	–	–	5	–	–	–
Glucose g/l	15	15	20	15	20	–	20	–	–	–	–	–
Glycerol (ml/L)	–	2.5	–	–		40	–	40	40	–	–	–
NaCl(g/L)	5	5	–	–	5	–	–	–	–	–	–	–
CaCO_3_(g/L)	1	1	–	–	–	5	5	–	–	5	–	–
NH_4_Cl(g/L)	–	–	–	–	–	4	4	–	–	4	–	–
K_2_HPO4(g/L)	–	–	1	–	–	–	–	–	1	–	–	–
KH_2_PO_4_(g/L)	–	–	0.1	–	–	–	–	–	0.1	–	–	–
Beef extract(g/L)	–	–	–	–	5	–	–	–	–	–	–	–
TSB (g/L)	–	–	–	–	–	–	–	–	–	–	30	–
Soybean casein digest broth (g/L)	–	–	–	–	–	–	–	–	–	–	–	30

^a^ Soybean meal [21]

^b^ Soybean meal-yeast extract glucose [22]

^c^ Tryptone-yeast extract [23]

^d^ Yeast extract medium [24]

^e^ Beef extract medium [21]

^f^Aminoglycoside production medium [25]

^g^ ryptic Soy Broth (TSB) [15]

^h^ soyabean casein digest broth

^l^ Absence of ingredient

### Determination of the antibacterial activity and microbial growth

To examine the antibacterial activity of the paromomycin produced by *S. rimosus* subsp. *paromomycinus*, the culture broth (after various incubation conditions) was centrifuged and sterilized by filtration using 0.22 μm pore size cellulose membrane filters (CHMLAB, Barcelona, Spain). The culture filtrate was bio-assayed against *Staphylococcus aureus* ATCC 25923 by using agar well diffusion technique [26]. The suspension (0.5 McFarland) of *Staphylococcus aureus* ATCC 25923 was uniformly and aseptically spread on Mueller Hinton agar surface (MHA, Difco, USA) and 150 μl of the culture filtrate were used to fill the wells. Plates were kept at 4–8 °C for at least 30 min to allow the diffusion of culture filtrate. The inhibition zone diameters were recorded after 24 h of incubation at 37 °C. Growth was determined using the viable count technique [27].

### Estimation of paromomycin

Calibration curve was constructed by plotting known concentrations of paromomycin (standard paromomycin from Sigma Aldrich) expressed as micrograms per ml versus the corresponding inhibition zone diameters (obtained using agar well diffusion technique) against *S. aureus* ATCC 25923 after incubation of plates at 37 °C for 24 h). Paromomycin concentrations were quantified from this standard calibration curve using the developed linear equation.

### Optimization of culture media

To determine the optimum media for growth and antibiotic production, seed culture was generated by a loopful of *S. rimosus* inoculated in 25 ml (TSB) contained in 250 ml Erlenmeyer flask and incubating at 28 °C and 200 rpm. After 72 h of incubation, flasks containing 25 ml of each media used for optimization listed above were inoculated with 5.5% *v*/v of this seed culture (Table 2). Flasks were incubated at 28 °C for six days and 200 rpm. Growth and antibacterial activity was determined at the end of the incubation period.

### Optimization of different environmental conditions

These experiments were carried out using the best chosen media and the optimum temperature and agitation rate were selected using the conventional one-factor-at-a-time method. Paromomycin production was tested after incubation at different temperatures (15, 20, 28, 32.5, 37 and 50 °C) and different agitation rates (150, 200 and 250 rpm). Other factors including, pH (represented by the coded variable A), inoculum size (represented by the coded variable B) and incubation period (represented by the coded variable C) were optimized by RSM using the statistical software package Design Expert v. 7.0 (Design Expert Software, Stat-Ease Inc., Statistics Made Easy, Minneapolis, MN, USA) (Table 3). The software also produces analysis of variance (ANOVA) for the suggested model [28].

**Table 3:** The factorial design runs in Design Expert for three selected levels of the three tested factors inoculum size, pH and incubation period

Run Order	pH (A)	Inoculum size (B, %)	Incubation period (C, days)
1	9	1	6
2	7.5	5.5	6
3	9	5.5	3
4	7.5	5.5	6
5	6	5.5	9
6	7.5	1	9
7	7.5	5.5	6
8	9	10	6
9	7.5	5.5	6
10	6	1	6
11	6	10	6
12	7.5	10	3
13	7.5	1	3
14	6	5.5	3
15	7.5	5.5	6
16	7.5	10	9
17	9	5.5	9
Factor	Name	Level (−1)	level (+ 1)
A	pH	6	9
B	inoculum size (%)	1	10
C	incubation period (days)	3	9

### Experimental confirmation test of RSM results

A group of optimum culturing conditions was produced using the statistical optimization function in the Design Expert software and Paromomycin produced from a novel shaking flask fermentation experiment using these optimal factors was measured and compared with the results obtained using conditions which was not optimized.

### Statistical and graphical investigations

All experiments were performed in triplicate and the values calculated and plotted are the means of triplicate results while error bars indicate the standard deviation of the data.

In the RSM tests, all the experiments were done in triplicate and the mean of three readings was documented. All data investigations, response surfaces, model diagnostic plots and ANOVA analysis were produced using Design Expert v. 7.0.

### Paromomycin synergism assays

The effect of different combinations of paromomycin with other antibiotics on the six selected MDR clinical bacterial pathogens was studied as an attempt to find effective antibiotic combinations to overcome multiple resistances exerted by the respective MDR clinical isolates as well as to increase antimicrobial susceptibility to paromomycin. The tested isolates included four MDR Gram negative isolates namely, *P. aeruginosa* (isolates PS4 and PS25), *K. pneumoniae* (isolate KP13), *E. coli* (isolate EC10) and two MDR Gram positive pathogens namely, methicillin- resistant *Staphylococcus aureus* (MRSA isolate S36) and methicillin- sensitive *Staphylococcus aureus* (MSSA, isolate S41). To study the possible synergistic effect of combining paromomycin with different antimicrobial agents, the checkerboard assay was used. The antibiotics (each alone) used in combination with paromomycin were ceftriaxone, ciprofloxacin, ampicillin/sulbactam, azithromycin, clindamycin and doxycycline. The MIC of each antimicrobial agent was first determined using the broth microdilution technique as recommended by the CLSI guidelines [29, 30]. The stock solutions and serial twofold dilutions of each antibiotic were set according to the recommendations of CLSI immediately prior to testing [30]. Concentrations ranging from four to eight times the MIC to at least l/8 to 1/16 times the MIC in the final panel were prepared in order to observe the occurrence and magnitude of synergism or antagonism. Checkerboard assay was done as described previously [31]. Briefly, a total of 100 μl of Mueller-Hinton broth was distributed into each well of the microdilution plates. The first antibiotic of the combination was serially diluted horizontally, while the second drug was diluted vertically. An inoculum equal to a 0.5 McFarland turbidity standard was prepared from each isolate in Mueller-Hinton broth. Each microtiter well was inoculated with 100 μl of a bacterial suspension to obtain a final inoculum of 5 × 10^5^ CFU/ml, and the plates were incubated at 37 °C for 24 h.

The resulting checkerboard has each combination of two antibiotics, where the wells that contain the highest concentration of each antibiotic at opposite corners. According to the CLSI, the MIC was defined as the lowest concentration of antibiotic that inhibited the growth of the organism completely as detected with unaided eye [30]. The sum of the fractional inhibitory concentrations (ΣFICs) was calculated according to the equation:

$$ \mathsf{\varSigma FIC}=\mathsf{FIC}\ \mathsf{A}+\mathsf{FIC}\ \mathsf{B} $$

Where FIC A is the MIC of drug A in the combination/MIC of drug A alone, and FIC B is the MIC of drug B in the combination/MIC of drug B alone. The combination is considered synergistic when the ΣFIC is ≤0.5, additive when ΣFIC is > 0.5 and ≤ 1, indifferent when the ΣFIC is > 1 and ≤ 4, and antagonistic when the ΣFIC is > 4 as described previously [32].

## Results

The effect of different factors on paromomycin production was studied. Such factors included the medium used for production, the incubation temperature, agitation rate, inoculum size, duration of fermentation process in addition to pH of production medium.

### Estimation of paromomycin

**Fig. 1:**
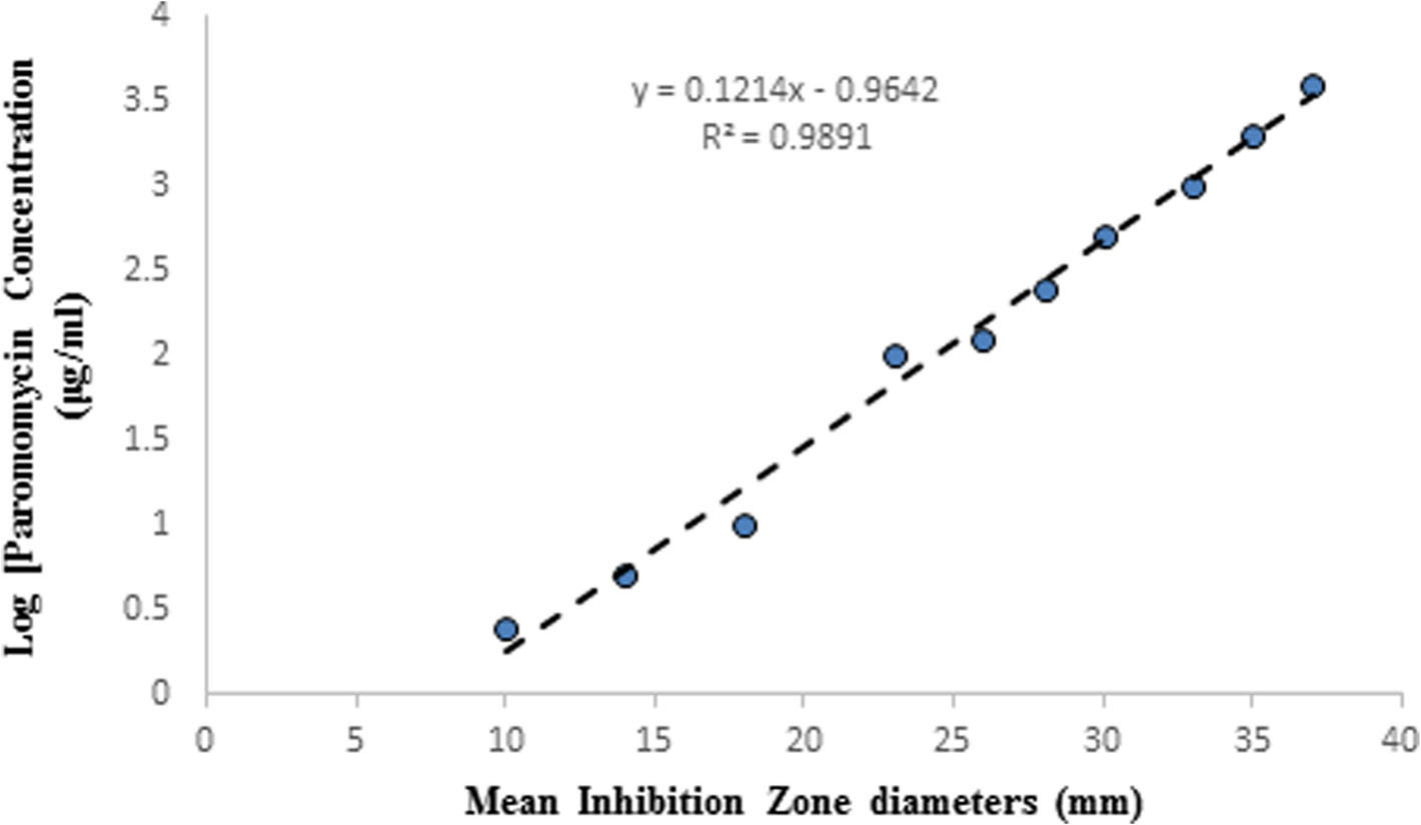
Calibration Curve of standard paromomycin antibacterial activity against *Staphylococcus aureus* ATCC 25923

Concentrations of paromomycin expressed as micrograms per ml was calculated using the developed linear equation Y (concentration of paromomycin in μg/ml) = 0.1214 X -0.9642 where X is inhibition zone diameter (mm) with *R*^*2*^ = 0.9891 as shown in Fig. 1.

### Effect of different production media

**Fig. 2:**
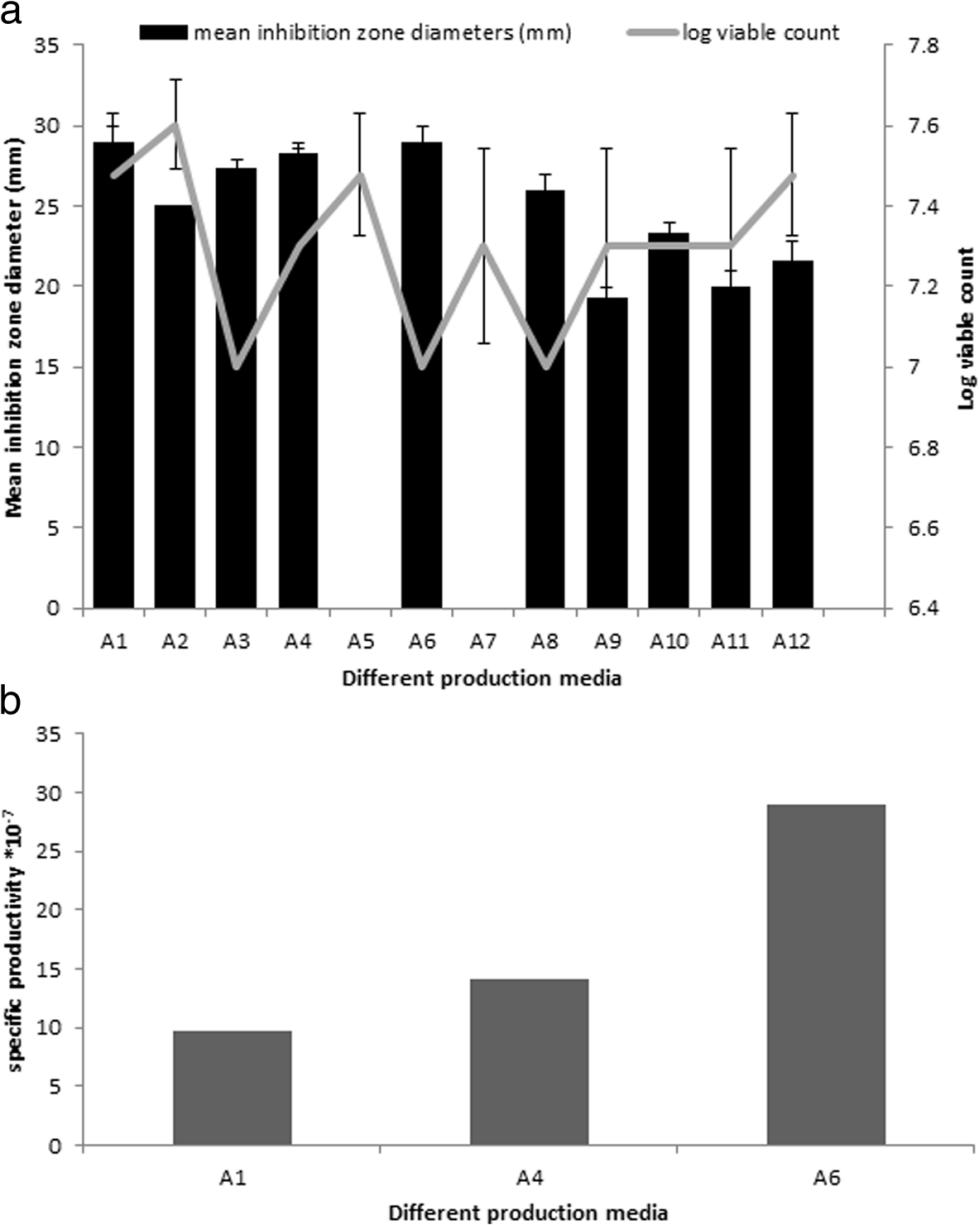
Effect of different culture media on the production of paromomycin and the growth of *S.rimosus* (**a**) and specific productivity of the three media having maximum paromomycin production (**b**)

As shown in Fig. 2a, soybean meal (A1) and aminoglycoside production medium (A6) gave the highest paromomycin production. This was followed by yeast extract medium (A4) then tryptone-yeast extract (A3). Other tested media (A2, A8, A9, A10, A11 and A12) produced much lower production while media A5 andA7 showed no production. Regarding cell growth, comparable viable cell counts was obtained with A2, A1, A5 and A12 which were higher than other tested media. The similar production in media A1, A4 and A6 led us to calculate the antibacterial activity of paromomycin per colony forming unit (specific productivity; Fig. 2b) to find out the optimum media for production. Aminoglycoside production medium (A6) showed the highest specific productivity so it was chosen to be the best medium for production.

### Effect of different environmental conditions

#### Effect of temperature on the paromomycin production

**Fig. 3:**
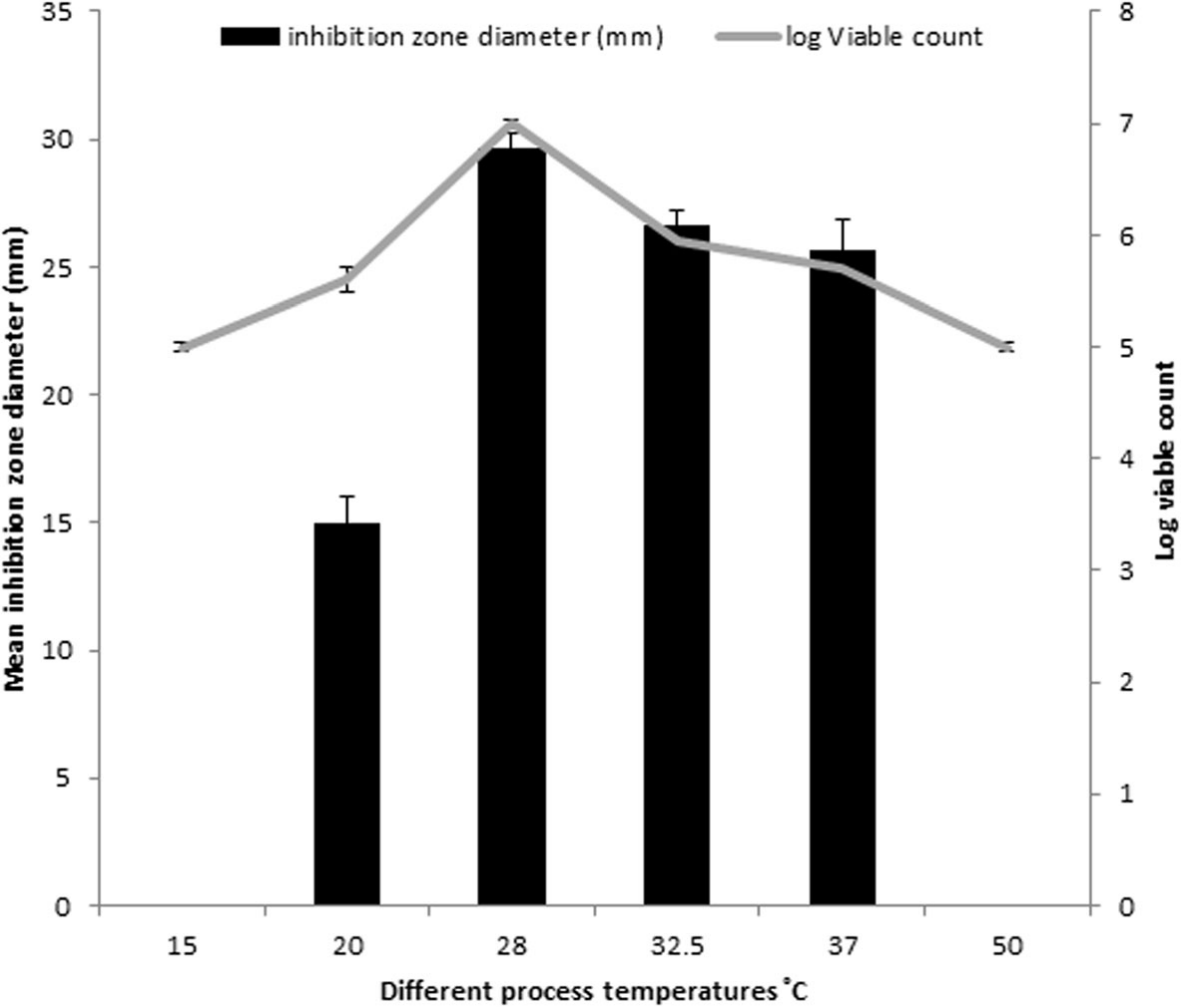
Effect of different temperatures on paromomycin production and bacterial growth

As shown in Fig. 3, the highest paromomycin production was obtained after incubation at 28 °C. Concerning bacterial growth, this temperature also showed the highest viable cell count. So, the optimum incubation temperature for paromomycin production was 28 °C.

#### Effect of agitation rate on the paromomycin production

**Fig. 4:**
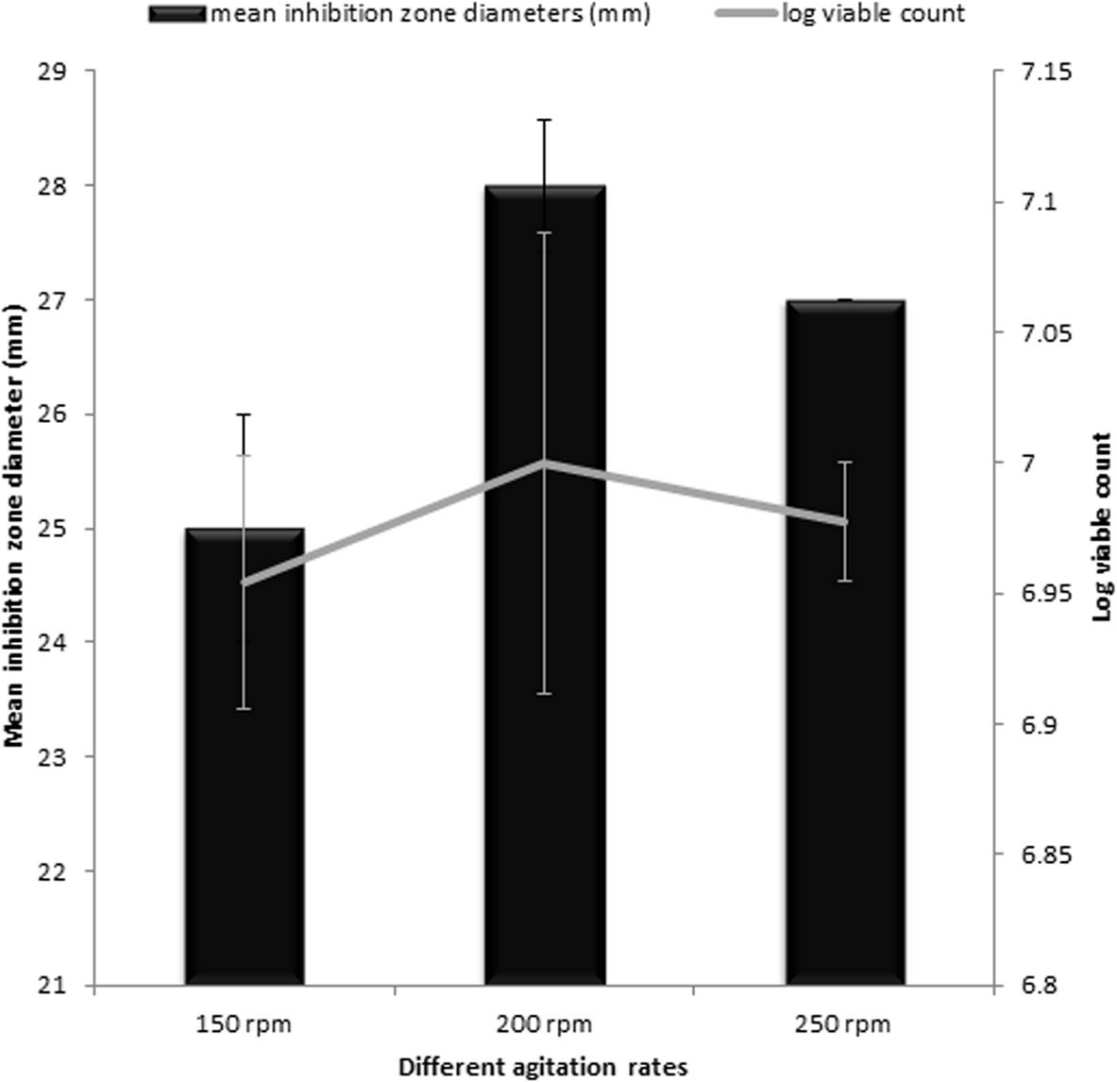
Effect of different agitation rates on paromomycin activity and bacterial growth

As shown in Fig. 4, the highest paromomycin production was obtained using an agitation rate of 200 rpm. Similarly, regarding bacterial growth, an agitation rate of 200 rpm showed the highest viable cell count. Accordingly, optimum agitation rate for paromomycin production was chosen to be 200 rpm.

#### Optimization of pH, inoculum size and incubation period using RSM

RSM, a set of statistical and mathematical techniques for modeling, is essential for optimizing conditions for the production of vital products such as antibiotics. Optimization studies aids in understanding the interactions among different environmental conditions. Multivariate experiments are constructed so that the number of experiments required in the optimization process decreased and the precision of the results obtained increased more than those obtained by univariate strategies [33]. This study deals with the optimization of the most important fermentation variables using RSM for the production of paromomycin from *S. rimosus* subsp*. paromomycinus.* The design of experiments was done by using Design Expert software.

The design, the experimental results, and the values predicted by the fitted equation obtained by the Design Expert software are shown in Table 4.Thefitted equation for the response is given by Eq. 1 for paromomycin activity (paro activity).

**Table 4:** The Box Behnken design with observed and predicted responses

Run Order	pH	Inoculum size (%)	Incubation period (days)	Observed response	Predicted response
1	9	1	6	14	17.6
2	7.5	5.5	6	28	26.8
3	9	5.5	3	0	2.0
4	7.5	5.5	6	28	26.8
5	6	5.5	9	34	34.5
6	7.5	1	9	27	25.1
7	7.5	5.5	6	28	26.8
8	9	10	6	22	22.1
9	7.5	5.5	6	28	26.8
10	6	1	6	20	22.4
11	6	10	6	25	23.9
12	7.5	10	3	0	−1.1
13	7.5	1	3	0	−4.1
14	6	5.5	3	0	3.3
15	7.5	5.5	6	27	26.8
16	7.5	10	9	26	28.1
17	9	5.5	9	30	29.3

Final Equation in Terms of Actual Factors:

Paromomycin activity = −47.6 –(0.36 * pH) + (2.4* inoculum size) + (19* incubation period) + (0.12* pH * inoculum size)-0.23* pH * incubation period-0.26* inoculum size^2^–1.1* incubation period^2^

ANOVA results are shown in Table 5. ANOVA confirms the suitability of the models and clarifies the significance of the various parameters that affects paromomycin production [33]. The Model F value of 43.12 and *p* value < 0.0001 for paromomycin production, proved the significance of the model. Moreover, C B^2^ and C^2^ were found to be significant model terms (Table 5) since *p* values less than 0.05 (α = 0.05) indicate that model terms are significant [28]. Low value of the coefficient of variation (CV) of 13.87% was gained which entailed the good quality steadfastness of the experimental values. The CV is an important value to show the level of precision with which the treatments are compared. The reliability of the experiment decreases as the CV value increases [35]., Moreover, the coefficient of determination R^2^ was 0.9710, showing that 97.10% of variability in the response could be demonstrated by the model. A Predicted R-squared (Pred. R^2^) of 0.8090 was achieved which was in good agreement with the Adjusted R-squared (Adj. R2) which was 0.9485. The predicted R-squared and the adjusted R-squared should be within 0.20 of each other [28]. In this model, a precision ratio of 20.490 was obtained suggesting an enough signal, since a ratio greater than 4 is usually required [36].

**Table 5:** Analysis of variance (ANOVA) for Response Surface Reduced Quadratic Model

Source	Sum of Squares	Df	Mean Square	F Value	*p*- value
Model	2282.42	7	326.06	43.12	< 0.0001
A-ph	21.13	1	21.13	2.79	0.129
B-inoculum size	18	1	18	2.38	0.1573
C-incubation period	1711.13	1	1711.13	226.3	< 0.0001
AB	2.25	1	2.25	0.3	0.5987
AC	4	1	4	0.53	0.4855
B^2^	117.54	1	117.54	15.55	0.0034
C^2^	383.17	1	383.17	50.67	< 0.0001
Residual	68.05	9	7.56		
Pure Error	0.8	4	0.2		
Cor Total	2350.47	16			

**Fig. 5:**
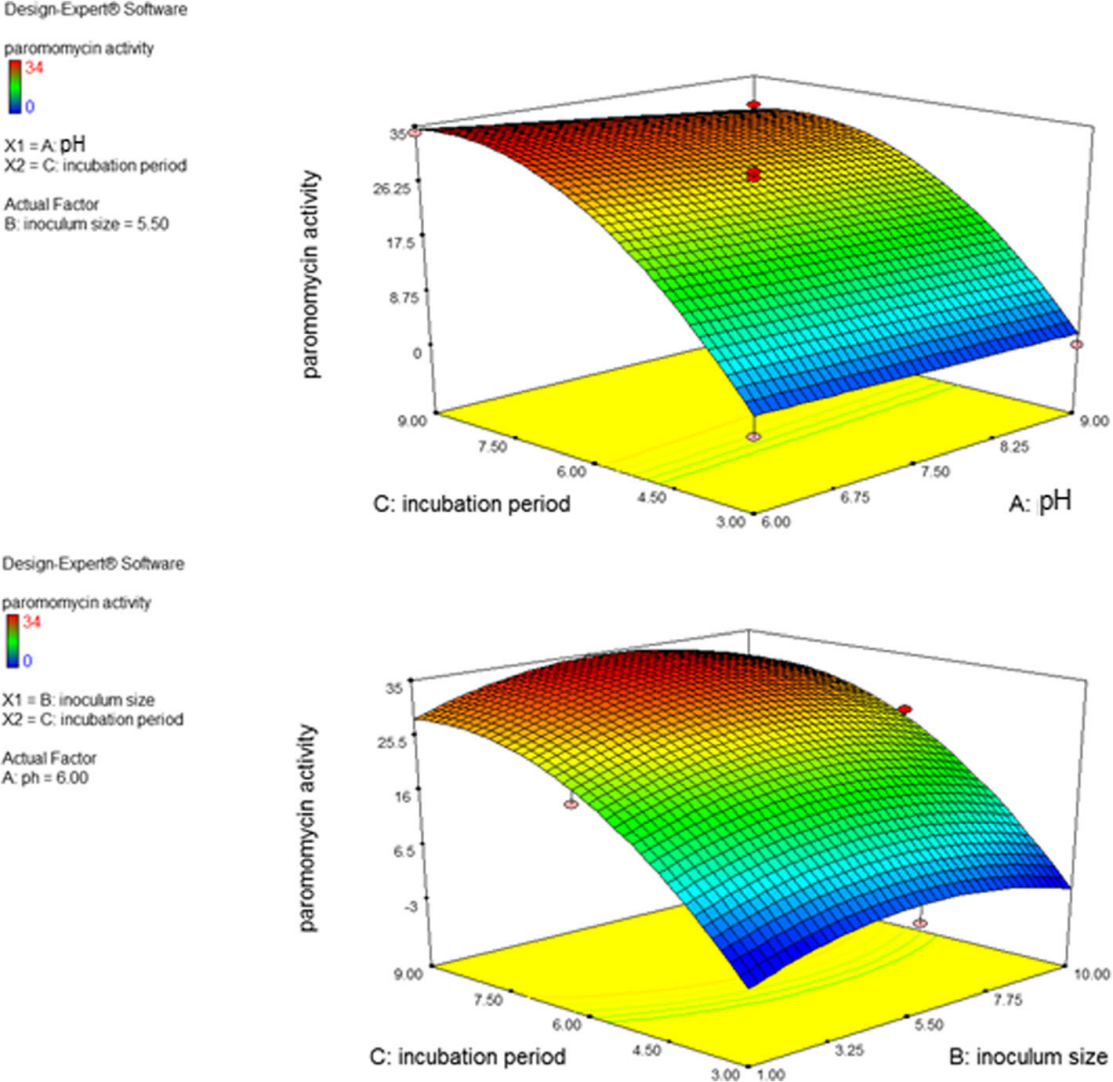
Three-dimensional (3D) surface plots for the effects of inoculum size, incubation time and pH on paromomycin activity (obtained from Design Expert software)

The three-dimensional (3D) plots between the input factors are delineated in Fig. 5. Optimum conditions for maximum paromomycin production were pH of 6, an inoculum size of 5.5% and an incubation period of 8.5 days.

#### Model diagnostics

To validate our models, graphical summaries were constructed as follows:

- Box Cox plots.

**Fig. 6:**
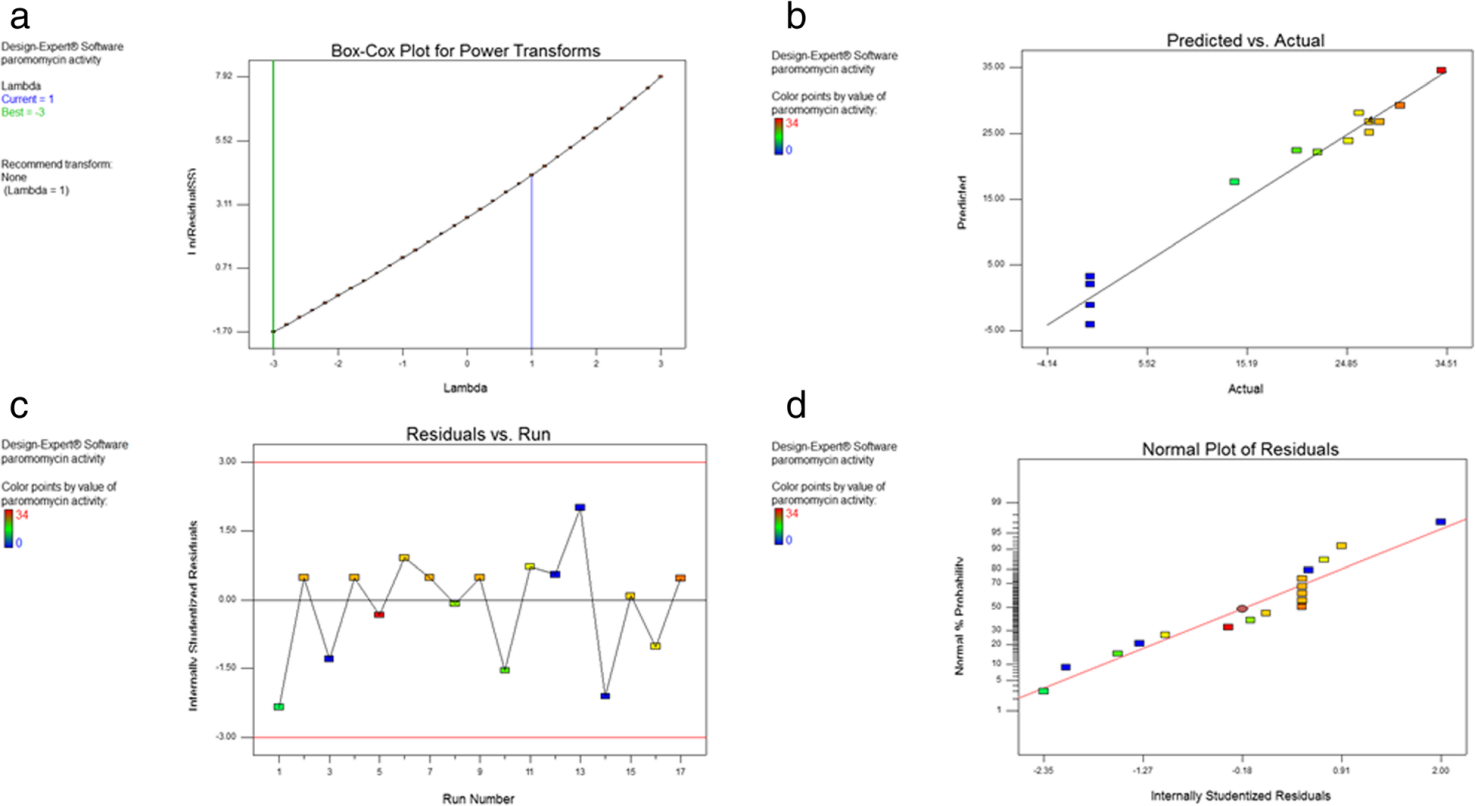
Model Diagnostics (Obtained from Design Expert software). **a** Box-Cox plot for Power Transforms; **b** Predicted vs. actual plot; **c** Residuals vs. Run plot; **d** Normal plot of Residuals

The Box–Cox plots showed that the model was sufficient and there was no need for additional transformation, where the existing lambda (lambda = 1) is within the 95% confidence range (Fig. 6a).

(b) Predicted vs. actual plot.

**Fig. 7:**
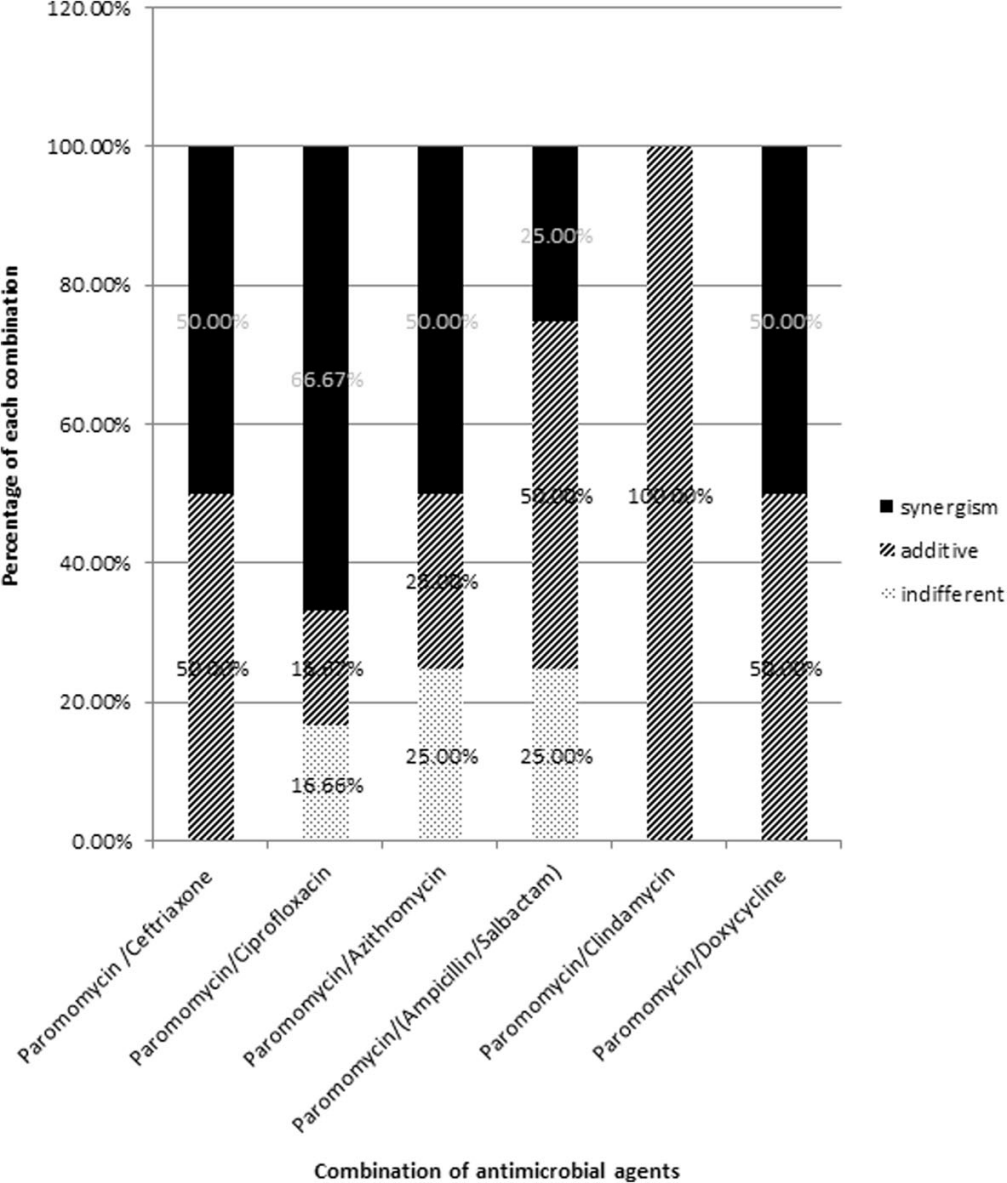
Effect of paromomycin combination with different antimicrobial agents

As delineated in Fig. 6b, the plot revealed that actual values were very close to the predicted ones.

(c) Residuals vs. run plot.

The points showed that the model fits the data as shown in Fig. 6c.

(d) Normal plot of the Residuals.

The plot indicated that the points track a straight line. These results revealed that the residuals follow a normal distribution, as shown in Fig. 6d.

The ANOVA results also showed that incubation period had a significant effect on paromomycin production. An increase in the incubation period generally enhances the growth and growth related activities of the organism up to a certain limit after which there could be a reduction in bacterial activity due to nutrient limitations. In this study, maximum paromomycin production was predicted to be obtained after 8.5 incubation days with inoculum size of 5.5% *v*/v and initial pH of 6.

#### Experimental confirmation test

Paromomycin production using the recommended optimal levels of the three factors (8.5 incubation days, 5.5% v/v and pH 6) resulted in an inhibition zone of 34.3 mm which was very close to that predicted by the model 34.7 which demonstrates the validity of the model. This value was equivalent to a standard paromomycin antibacterial activity of 1.584 mg/ml. obtained from the calibration curve done on the standard paromomycin. As delineated in Additional file 1 Figure S1, the optimal conditions used produced a 4.4-fold increase in paromomycin production as compared to that produced by using the optimized culture media alone and a 14-fold increase in paromomycin production as compared to that obtained using either un-optimized culture conditions or culture media.

### Paromomycin combinations with other antimicrobial agents

The MICs of the mentioned antimicrobial agents in the selected isolates, determined by the broth microdilution method, are shown in Table 6.

**Table 6:** The MICs of the antimicrobial agents in the selected isolates

Isolate	Species		MIC of different antimicrobial agents (μg/ml)/Susceptibility					
		paromomycin	Ceftriaxone	Ciprofloxacin	azithromycin	ampicillin/ sulbactam	clindamycin	doxycycline
PS4	*P. aeruginosa*	256	1024	32	512	170.67/85.3		–
PS25	*P. aeruginosa*	64	256	16	16	42.67/21.33		–
KP13	*K. pneumoniae*	1	128	1024	64	21.33/10.67		–
EC10	*E.coli*	4	256	0.5	64	10.67/5.33		–
SA36	MRSA	2048	1024	128	–	–	0. 5	1
SA41	MSSA	2048	16	128	–	–	1	2

Three different effects on paromomycin activity were achieved by the tested combinations including: synergistic (S; ΣFIC is ≤0.5), additive (A; ΣFIC is > 0.5 and ≤ 1), and indifferent effects (I; ΣFIC is > 1 and ≤ 4), as shown in Tables 7 & 8. Likewise, no antagonistic effects were observed with any of the combinations of antimicrobial agents studied. Percentages of different effects (A, I or S) accompanying different antibiotic combinations are shown in Fig. 7.

**Table 7:** MICs of different antimicrobial agents among the isolates selected for synergism assays

Isolate	Species		MIC of different antimicrobial agents (μg/ml)/Susceptibility					
		paromomycin	Ceftriaxone	Ciprofloxacin	azithromycin	ampicillin/ sulbactam	clindamycin	doxycycline
PS4	*P. aeruginosa*	256	1024	32	512	170.67/85.3		–
PS25	*P. aeruginosa*	64	256	16	16	42.67/21.33		–
KP13	*K. pneumoniae*	1	128	1024	64	21.33/10.67		–
EC10	*E.coli*	4	256	0.5	64	10.67/5.33		–
SA36	MRSA	2048	1024	128	–	–	0. 5	1
SA41	MSSA	2048	16	128	–	–	1	2

**Table 8:** Effects of different antimicrobial combinations on Paromomycin activity among selected isolates

No.	Species	Antimicrobial combination	Concentrations^a^(μg/ml)	ΣFIC^b^	Interpretation
PS4	*P .aeruginosa*	Paromomycin/ceftriaxone	32/256	0.375	S
		Paromomycin/ciprofloxacin	128/1	0.53125	A
		Paromomycin/azithromycin	8/256	0.28125	S
		Paromomycin/ampicillin-sulbactam	4/(85.33/42.67)	0.51625	A
PS25	*P. aeruginosa*	Paromomycin/ceftriaxone	32/64	0.75	A
		Paromomycin/ciprofloxacin	16/1	0.3125	S
		Paromomycin /azithromycin	4/16	1.03125	I
		Paromomycin/ampicillin-sulbactam	8/(21.33/10.67)	0.625	A
KP13	*K. pneumoniae*	Paromomycin/ceftriaxone	0.5/2	0.515625	A
		Paromomycin/ciprofloxacin	0.25/256	0.5	S
		Paromomycin/azithromycin	0.25/32	0.75	A
		Paromomycin/ampicillin-sulbactam	1/(0.67/0.33)	1.03125	I
EC10	*E.coli*	Paromomycin/ceftriaxone	1/16	0.3125	S
		Paromomycin /Ciprofloxacin	1/0.125	0.5	S
		Paromomycin/azithromycin	0.25/0.25	0.5	S
		Paromomycin/ampicillin-sulbactam	0.5/(1.33/0.67)	0.1875	S
PS36	MRSA	Paromomycin/ceftriaxone	256/16	0.140625	S
		Paromomycin/ciprofloxacin	256/8	0.1875	S
		Paromomycin/clindamycin	512/0.25	0.75	A
		Paromomycin/doxycycline	64/0.5	0.53125	A
PS41	MSSA	Paromomycin/ceftriaxone	512/8	0.75	A
		Paromomycin/ciprofloxacin	32/128	1.015625	I
		Paromomycin/clindamycin	1024/0.125	0.625	A
		Paromomycin/doxycycline	64/0.5	0.28125	S

^a^ Concentrations of the respective antimicrobials agents at which the lowest value of ΣFIC was achieved.

^b^ΣFIC, the sum of the fractional inhibitory concentrations of the combined antimicrobial agents.A, additive effect; I, indifferent effect; S, synergistic effect

## Discussion

The culture medium components and conditions of the organisms often influence the production of bioactive metabolites [37]. An attempt was made in the present study to optimize nutritional and environmental conditions leading to higher paromomycin production by the studied isolate. For the production culture, different culture media were tested. Agitation rate and incubation temperatures for fermentation process were also studied. Using Response Surface Methodology, other factors including, pH, inoculum size and incubation period were optimized using the statistical software package Design Expert v. 7.0 (Design Expert Software, Stat-Ease Inc., Statistics Made Easy, Minneapolis, MN, USA).At the end of each run the antimicrobial activity of paromomycin was bioassayed against *Staphylococcus aureus* ATCC 25923. Also viable count technique was used to measure the bacterial growth.Different production culture media were used for tested isolates as described previously. Our results showed that soybean meal (A1) and aminoglycoside production medium (A6) gave the highest paromomycin production and this production was several folds higher than other tested media. This was followed by yeast extract medium (A4) then tryptone-yeast extract (A3) where there was a sharp decrease in activity. Other tested media (A2, A8, A9, A10, A11 and A12) produced much lower production while media A5 and A7 showed no production. Regarding cell growth, comparable viable cell counts was obtained with A2, A1, A5 and A12 which were higher than other tested media. Calculation of the antibacterial activity of paromomycin per colony forming unit was a must to find out the optimum media for production as media A1, A4 and A6 have similar production. Results showed that aminoglycoside production medium (A6) was the best medium for production as it showed the highest specific productivity.

The effect of incubation temperature on biomass and paromomycin production was studied. The paromomycin production was found to be maximum at incubation temperature 28 °C. Regarding Cell growth, optimum growth was obtained at incubation temperature 28 °C.This finding is in accordance with that obtained by Singh et al., 2014 where they found that 28 °C was found to be optimum for highest growth as well as maximum antimicrobial agent production by *Streptomyces sannanensis* strain SU118 [38].Increasing rotation speed to 200 rpm resulted in maximum paromomycin production and the highest growth. After optimizing the culture media for paromomycin production, it was necessary to optimize the environmental fermentation conditions. This was done using RSM which is beneficial mathematical and statistical tool; it is useful for finding experimental design for illuminating the relativeness among the factors and the best combination of parameters and also it predicts responses [39]. In this study, we undertook a complete factorial design for optimization of paromomycin production by *Streptomyces rimosus* subsp*. paromomycinus* NRRL 2455. The design of experiments, modeling, data analysis by ANOVA, generation of response surfaces (and their respective contour plots) and diagnostic plots. The 3D response surface curves and their respective contour plots give full description about the interactions between two parameters and enable a simple estimation of the optimal experimental conditions [35]. From these plots and using numerical optimization function, optimal conditions for maximum paromomycin production were found to be inoculum size 5.5% *v*/v, pH of 6 and incubation Period of 8.5 days. Paromomycin production using the recommended optimal levels of the three factors resulted in an inhibition zone of 34.3 mm which was very close to that predicted by the model 34.7 which demonstrates the validity of the model. This value was equivalent to a standard paromomycin antibacterial activity of 1.584 mg/ml. obtained from the calibration curve done on the standard paromomycin.ANOVA was used to investigate the results; it also verified the suitability of the models and described the significance of the factors on paromomycin production. The *P value* decides the significance of the results [40]. ANOVA results for the models revealed that the model equations generated were significant and could adequately be used to describe the paromomycin production. The CV represents the accuracy of the comparison between the treatments. Increasing the CV value indicates that the experiment reliability decreases [35]. The great reliability of the experimental values was revealed by the low CV value in this study.To validate the generated models, it was necessary to construct graphical summaries for case statistics. The Box–Cox plot not only guides for picking the correct power law transformation but also states whether the response needs to be measured on a different scale or not. No transformation is recommended since the current lambda is within 95% confidence range. The predicted versus actual plot is used quantitatively to compare the results of the experimental values of the response with that of the predicted values from the generated models. The residuals versus the experimental run order are represented by the residual versus run plot [34]. The resulted plots constructed in this study prove the validity of the model. To study the possible synergistic effect of combining paromomycin with different antimicrobial agents, the checkerboard assay was used. The MIC of each antimicrobial agent (ampicillin/sulbactam, ceftriaxone, ciprofloxacin, azithromycin, clindamycin and doxycycline).was first determined using the broth microdilution technique as recommended by the CLSI guidelines [28]. According to the CLSI guidelines, MIC is the lowest concentration of antimicrobial agent that inhibit completely the growth of microorganism as inspected by unaided eye [29] Three different effects on paromomycin activity were achieved by the tested combinations including: synergistic (S; ΣFIC is ≤0.5), additive (A; ΣFIC is > 0.5 and ≤ 1), and indifferent effects (I; ΣFIC is> 1 and ≤ 4). The tested combinations showed synergistic effect on paromomycin activity on most of the tested isolates (45.83%), additive effect in 41.67% of them and indifferent in 12.5%. all these combinations had considerable synergistic and additive effects which are desirable for these drug resistant isolates. The concentrations of some drugs used in this study were of clinically unachievable levels and therefore should be considered as purely theoretical. Therefore, additional in vivo studies to assess the clinical efficacy of combinations are needed. Moreover, antimicrobial combination must be based on sound knowledge about the effect of two or more drugs in combination to avoid possible untoward effect like antagonism. We are expanding in future this investigation to include additional species. Standard in vitro methods do not consider changes of antibiotic concentrations over time during combination therapy. Concentrations studied are defined according to bacterial sensitivity (fractions of MIC). These results revealed that combination therapy of antimicrobials should be considered for the treatment of serious Gram-negative and Gram-positive infections [41].The synergistic effect of in vitro study seems to be the best therapeutic choices; however additional clinical studies should be done to verify that the clinical response is also significant.

## Conclusion

It was shown that RSM is suitable for optimization of paromomycin production by *S. rimosus*NRRL2455. Application of RSM helped us to reach optimal culture conditions with a minimum number of experimental trials. Maximum paromomycin production was obtained using the aminoglycoside production medium containing 40 ml/L glycerol as the carbon source and 30 g/L soy bean meal as the nitrogen source. The optimum conditions were found to be a temperature of 28 °C, agitation rate of 200 rpm, inoculum size of 5.5% *v*/v, pH of 6 and an incubation period of 8.5 days. This resulted in about 14 fold increase in paromomycin production reaching 1.584 mg/ml which is greater than most of the comparable data cited in the literature. Antibiotic combinations of paromomycin with showed synergistic effect with most of on most of the tested isolates (45.83%), additive effect in 41.67% of them and indifferent in 12.5%.Therefore, *S. rimosus* subsp. *paromomycinus* NRRL 2455 can be considered to be a promising bacterial isolate for further industrial exploitation and Paromomycin Synergism with other antimicrobials make it the drug of choice for the treatment of diseases caused by multidrug resistant pathogens.

## Additional file

Additional file 1: **Figure S1.** Comparison of growth and paromomycin activity by *S .rimosus* using optimized and un-optimized conditions. (TIF 29 kb)

## Abbreviations

ATCC: American type culture collection
FIC: Fractional inhibitory concentrations
MHB: Mueller hinton broth
MIC: Minimum inhibitory concentration
NRRL: Northern regional research laboratory
RSM: Response surface methodology
TSB: Tryptic soy broth
