# Supplementary figures and images for: Paromomycin production from Streptomyces rimosus NRRL 2455: statistical optimization and new synergistic antibiotic combinations against multidrug resistant pathogens
Source: BMC Microbiol. 2019 Jan 18;19:18. doi: 10.1186/s12866-019-1390-1 (PMC6339272; doi:10.1186/s12866-019-1390-1)

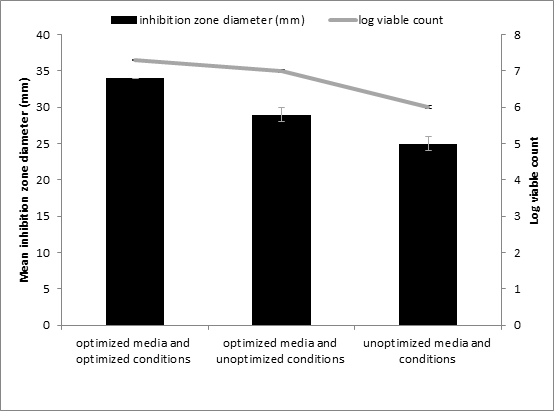

Supplement: Supplementary file 1 — Figure S1. Comparison of growth and paromomycin activity by S .rimosus using optimized and un-optimized conditions. (TIF 29 kb) [file 12866_2019_1390_MOESM1_ESM.tif]
